# A Study in Stillness and Symmetry

**DOI:** 10.3201/eid2510.AC2510

**Published:** 2019-10

**Authors:** Byron Breedlove

**Affiliations:** Centers for Disease Control and Prevention, Atlanta, Georgia, USA

**Keywords:** art science connection, emerging infectious diseases, art and medicine, about the cover, Robert Lee Kocher, Two Birds, a study in stillness and symmetry, birds, common grackle, Quiscalus quiscula, viruses, avian influenza, influenza virus, bacteria, fungi, respiratory infections, fungal infections, fungal pathogens, bacterial infections, bacterial pathogens, zoonoses, North America

**Figure:**
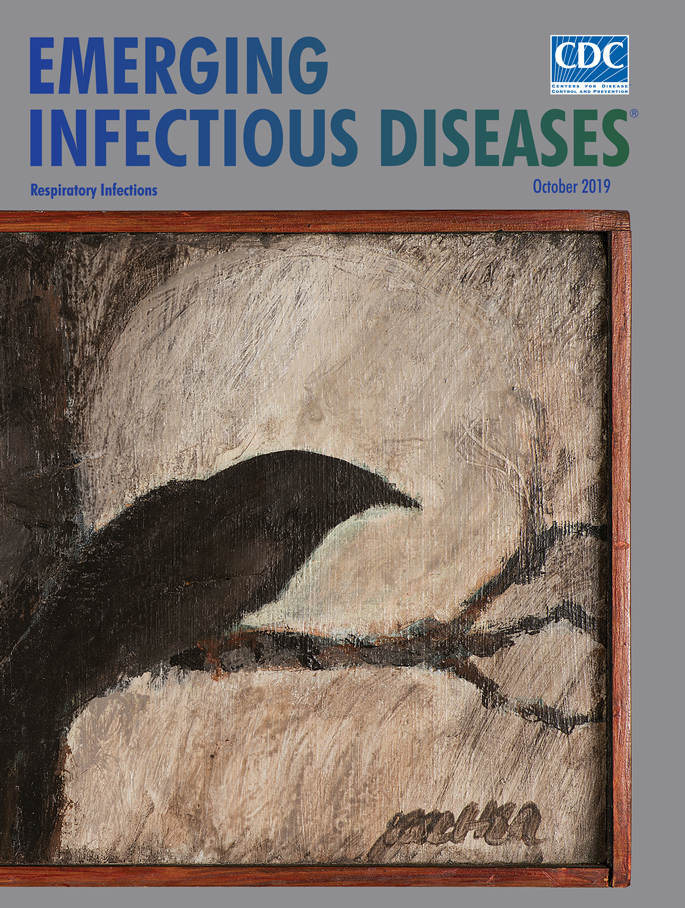
**Robert Lee Kocher (1929–), *Two Birds* (1959).** Oil on wood, 12 in × 7.75 in/30.5 cm × 19.7 cm. Image used by permission of the artist. Private collection, Fayetteville, Georgia, USA. Photography by James Gathany.

With an MA degree in art from the University of Missouri, Robert Lee Kocher pondered a career illustrating medical textbooks. Instead, his passion for painting led him to pursue a course that allowed him full immersion in art as both an academic and an artist. Kocher served as chair of the art department at Coe College in Cedar Rapids, Iowa, USA, where he is the Marvin D. Cone Professor of Art, Emeritus. Cone, who preceded Kocher at Coe College, was a close friend of a famous painter associated with Cedar Rapids, Grant Wood (P. Kocher, pers. comm., email, 2019 Aug 4).

Throughout his career, Kocher found opportunities to explore myriad styles and formats. Many of his works are drenched in color, whereas others feature deliberately limited palettes. His versatility is underscored by the contrast between his vivid abstract works overlaid with petroglyphs that he created in New Mexico and his more subdued rendering of a row house near the South Carolina coast. Kocher sometimes painted directly on wood instead of traditional surfaces, using the textures from the wood as another distinctive element.

*Two Birds*, appearing on this month’s cover, is one of Kocher’s more somber paintings. The artist depicts a pair of common grackles, (*Quiscalus quiscula*), perched on a tree branch, visible from a window in his late mother’s home. The distinct symmetry of dark and light tones instills a palpable sense of stillness and quiet. He matches the dark colors of the birds and the tree but varies their textures. Kocher’s grackles are nearly all black: purple, green, and blue iridescence is muted in the filtered sunlight of an overcast winter’s afternoon.

Native to North America, grackles forage in farm fields, rural pastures, suburban lawns, cattle feedlots, and marshes. Their song is a cacophonous crackling and high-pitched screech that the National Audubon Society guide likens to the sound made by a rusty hinge. In his poem “The Grackle,” Ogden Nash does not flatter the species, writing, “I cannot help but deem the grackle / an ornithological debacle.”

Kocher’s painting, however, offers a different perspective on this common bird. Kocher probably looked out that window many times before he noticed the symmetry and contrast that prompted him to paint *Two Birds*. His grackles are subdued, claws tightly clenched as they huddle, their curved silhouettes edged by the cold Iowa winter. The bare tree branches that bisect the lower part of the painting extend beyond the edges of the painting, like unclenched claws grasping for warmth in cold sunlight. Kocher focuses on a pair of grackles, but his painting serves as a quiet reminder that birds are our ubiquitous neighbors.

Indeed, tallying the number of individual birds on Earth is a challenging endeavor. Some bird experts suggest an upper range of 200 to 400 billion, or 40 to 60 birds for every human. Birds are incredibly diverse—a recent study suggests there are more than 18,000 species—and very mobile. The interconnections among birds, humans, domestic animals, and wildlife are intricate and of concern to public health. 

Wild and domestic birds carry various emerging and reemerging pathogens, including some that can be transmitted to humans. For example, soils with large accumulations of bird droppings may expose humans to potentially infectious fungal pathogens such as *Cryptococcus neoformans* or *Histoplasma capsulatum*, although the human respiratory infections caused by those pathogens—cryptococcosis or histoplasmosis—are rare. Avian influenza A viruses have sporadically caused severe, sometimes fatal, infections in humans. *Chlamydia psittaci* has caused rare zoonotic respiratory infections.

A safe and healthy world depends on animal, environmental, and human health. Birds are found in virtually every ecosystem, so studying their worldwide population numbers provides compelling evidence for gauging the overall health of our environments. Because pathogens can mutate and gain the ability to spread among people, scientists must vigilantly monitor avian populations for telltale signs that could signal a spillover event. Not to do those things would be, to revisit Nash’s poem, a very serious ornithological debacle.
